# Reforming for trust and professionalism in municipal healthcare services: implications for human resource management

**DOI:** 10.1186/s12913-023-10006-8

**Published:** 2023-09-14

**Authors:** Olaug Øygarden, Martin Nøkleberg, Leif Jarle Gressgård

**Affiliations:** 1 Division of Health and Social Sciences, NORCE Norwegian Research Centre, Prof. Olav Hanssensvei 15, N-4021 Stavanger, Norway; 2 Department of welfare and participation, Western Norway University of Applied Sciences, Postbox 7030, N-5020 Bergen, Norway; 3 Division of Health and Social Sciences, NORCE Norwegian Research Centre, Nygårdsgaten 112, N-5008 Bergen, Norway

**Keywords:** Home-based municipal healthcare services, Organizational model, High-involvement work systems, Professionalism, Trust reform

## Abstract

**Background:**

Many countries face an increasing demand for home-based healthcare services, and consequently experience a mismatch between expectations and available financial and human resources. It is therefore important to utilize human resources more efficiently, while at the same time offer jobs that attract the professionals they need. This article reports a study of the development and piloting of a new organizational model for home-based healthcare services in a Norwegian municipality, which addresses the need to provide efficient services and enhance trust and professionalism within healthcare services by improving work autonomy and involvement of employees.

**Methods:**

The research project this article draws its empirical material from was commissioned by the municipality piloting the new organizational model and executed in collaboration with the municipality based on an evaluative trailing research (ETR) design. The data consists of interviews with key personnel and knowledge exchange between researchers and the involved actors in the pilot project. 20 semi-structured interviews involving a total of 34 informants were conducted.

The analysis emphasises how different employee groups and management perceived and experienced various aspects of the work situation, as they were introduced to working and managing within the new organizational model. The aim is to shed light on how these employees and managers feel about it, interpret it, and respond to it.

**Results:**

Overall, the results indicate that the model holds potential for realizing the benefits it aims for. However, there were also challenges that need resolving for the model to fulfil this potential. Central elements include clarification of roles and responsibilities for employees and managers, competence specification and development, and development of structures for inter-professional cross-team collaboration and information provision.

**Conclusions:**

Trust reform initiatives may be a strategy for fostering high-involvement work systems. To achieve this, sufficient attention must be paid to ensuring structures for information exchange and knowledge development in the early phases of implementation, or preferably prior to implementation. The theoretical model applied in this study could potentially be a useful managerial tool in preparing for and implementing trust reforms in healthcare services.

**Supplementary Information:**

The online version contains supplementary material available at 10.1186/s12913-023-10006-8.

## Background

Norwegian municipalities face an increasing demand for healthcare services, both in quantitative terms and in terms of increased expectations for more coherent, integrated care [1]. At the same time, municipalities expect financial resources and access to qualified personnel to diminish in the coming years [2]. This mismatch between demands, expectations and available financial and human resources means that municipalities face a challenge of utilizing human resources more efficiently and offering jobs that attract the professionals they need. In order to amend this challenge, various reform initiatives across the Nordic countries have been implemented as part of what can be characterized as a trust agenda, which seeks to pave the way to enhance trust and professionalism among healthcare services [3–5].

Taking this as its point of departure, this paper presents findings from a qualitative case study following the development and piloting of a new organizational model for municipal home-based healthcare services in Norway. The specific model examined in this paper was implemented as part of a municipal trust and professionalism reform, and the municipality aimed for (i) optimal utilization of registered nurse human resources, (ii) increased autonomy and independent responsibility for healthcare workers^1^, as well as more professionally fulfilling work for registered nurses, (iii) closer integration of different professional groups, and (iv) service quality improvement.

**Fig. 1:**
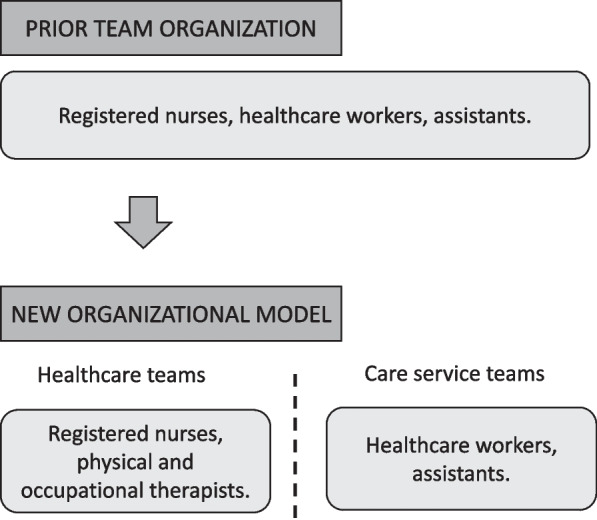
Team organization prior to and after reorganization

The implementation took place in a large municipality. The municipal department of home-based healthcare services is in charge of several units, each with their own manager and serving patients in distinct geographic zones. The department and its underlying units collaborate with other parts of the municipal healthcare services. Before reorganization, registered nurses, healthcare workers and assistants were organized as multidisciplinary units and sub-units. The new organizational model, which was piloted in one of these units, allocates tasks, end users and employees into two separate types of sub-units, referred to by the municipality as teams, as illustrated in Fig. 1 below. *Healthcare teams* consist of registered nurses and physical and occupational therapists, either as formal team members or still employed by a different municipal department (as before reorganization), but in close and frequent collaboration with the registered nurses. End users in need of services that formally require the competence of these groups, and the tasks involved in providing these services, were allocated to the healthcare teams*. Care service teams* consist of healthcare workers and assistants. End users in need of care services not requiring formal competence at the level of registered nurses are allocated to the care service teams. Patients are allocated to teams according to a set list of criteria, detailing which tasks can be performed by healthcare workers or assistants in care service teams, and tasks that require the competence of registered nurses in healthcare teams. Some patients have complex needs requiring visits from both types of teams. The responsibility for these patients is allocated to the team that will perform the most tasks in their home, and staff from the other team will visit to perform the additional tasks that are needed. Ideally, the teams collaborate and meet to discuss these patients to ensure coherence, and the responsibility for the patient may shift from one team to another as their needs change.

While tasks had been allocated to staff according to the specific competence of the different professional groups in the existing model as well, this division was challenged by day-to-practical and logistical issues. Particularly for registered nurses, this meant that tasks not requiring their level of competence was often delegated to them. The new team model was intended to draw up a clear, consistent and lasting division of tasks and responsibilities between the different professional groups. It was also intended to create a larger and more robust professional community for the nurses, as well as giving healthcare workers more independence and highlighting their unique competencies.

With the new organizational model, the municipality envisioned benefits at three interrelated levels: for *employees* in the form of work design characteristics, which impact behavioral, attitudinal, role perception and well-being outcomes, for *service users* in the form of improved experience of service quality, and for *the municipality* in the form of more efficient utilization of human resources in relation to the population’s need for services.

Reforming and reorganizing for trust and professional autonomy within public services is a relatively new phenomenon [6, 7]. Few studies have investigated links between such reforms and concepts from the human resource management (HRM) literature. In many ways, the aims of trust based organizational models are similar to what the HRM literature calls high-involvement work processes (HIWPs). When such work processes are supported by investments in human capital, they form part of high-involvement work systems (HIWSs) [8]. The present paper seeks to engage with these conceptual perspectives by investigating whether trust reform initiatives may be a strategy for fostering HIWSs, while also steering clear of the tensions these embody. More specifically, we provide an empirical analysis, from the Norwegian context, of how different employee groups and management perceived and experienced the dimensions of HIWSs, as they were introduced to working and managing within the new organizational model for municipal home-based healthcare services. The aim is to shed light on how these employees and managers feel about it, interpret it, and respond to it.

This paper is organized as follows. It begins with a review of the relevant literature on the rationale of trust reforms and presents the theoretical properties of HIWPs and HIWSs, and how trust-based reforms interlink with these concepts drawn from the HRM literature. Next, the research methods and design are accounted for. This is followed by a presentation of the empirical analysis, emphasising employees’ and managers’ perceptions of changes to their work roles in terms of autonomy and involvement following the new organizational model. In closing, we discuss how achieving HIWSs within the context of home-based healthcare services through reforming for trust and professionalism requires going beyond simply implementing more trust in and space for professional autonomy, as it also depends on developing systemic work elements.

## Theoretical background

### Reforming for trust-based management of healthcare services

Starting in the late 1970s, widespread changes were introduced to public sectors, including healthcare services, across western countries. Healthcare system reforms grounded in New Public Management (NPM) have been motivated by a desire to improve service efficiency and effectiveness, responsiveness to the public and managerial accountability, and to reduce public spending [9, 10]. A common theme in the understanding of NPM reforms is that managerial and economic principles have gained in importance vis-à-vis professional principles of organizing along lines of professional hierarchies and power, with administration being merely a support function [9, 11–13].

The wider reform literature has recognized a set of challenges created by NPM measures. These include fragmentation of services, coordination issues and inefficiencies because organizational units have been split up and made accountable only for their own results, meaning they work towards their goals in isolation from other units [14], and increased costs resulting from the need to coordinate services across a multitude of sub-units and management levels [15, 16]. Policies and reforms have therefore started turning towards reintegration and coordination, a development referred to as Post-NPM, whole-of-government, joined-up government or new public governance (NPG) [13, 16]. This development also recognizes that policy formulation is increasingly carried out by a vast number of formal and informal institutions and processes, which involves network governance responses [17, 18]. Thus, these processes form the background for a trend towards more multidisciplinary organizational structures [19, 20], and, in the context of healthcare services, a focus on integrated care [21].

Increasingly, the erosion of professional autonomy is also discussed as a problematic effect of managerialist and NPM inspired reforms, resulting in a call for trust and professionalism reforms within public sector organizations generally, and healthcare services more specifically. As a response, several reform initiatives and innovation processes are currently taking place at local, municipal levels across Scandinavian countries [4–7, 22–24]. Research and literature on the implementation of innovation in the public sector, its opportunities, and barriers that are often encountered has increased in the past years [25, 26]. However, as trust reform is a relatively new phenomenon, there are few studies explicitly addressing trust in relation to the management and organization of public services and the effects of trust and professionalism reforms [3, 6]. Generally, the reforms are assumed to foster increased innovation, engagement, efficiency and service quality, but this has not been examined longitudinally.

According to Bouckaert [27] trust can be more or less present in three different relationships around and within the public sector. First, citizens may trust the sector, secondly, the sector may trust the citizens, and thirdly, there may be trust internally between the different actors in the sector. In this paper, we report on a reform initiative aiming for increased utilization of professional competence, in parallel with increased responsibilities for employees and increased employee autonomy. The initiative can therefore be linked to the trust in relations internal to the public sector, and specifically to the trust in employees by management, as a model implying increased, professional decision latitude for employees is implemented. The trust and professionalism reform initiated by the municipality, as well as the pilot project we examine in this paper, may therefore be seen as part of the movement away from NPM, where internal trust is based on detailed control and performance management, and towards post-NPM or NPG ideals of basing trust on the idea of partnerships [27].

Bentzen [6], having studied the implementation of trust reform in the municipality of Copenhagen, Denmark, argues that these ideals imply a form of management and organization which she calls trust-based management. The Copenhagen trust reform, which included parts of the municipal healthcare services, aimed for increasing trust in the professional judgment of both managers and employees, and by doing so, reducing costs, enabling more time spent on core service provision tasks, and improving both service quality and employee job satisfaction. Within the home-based healthcare services, this implied replacing a system of strict and detailed time allocations for work tasks with a system of allocating time blocks for each home visit, within which employees had more freedom to flexibly perform their work. The municipality of Oslo, Norway, has also implemented similar pilot projects within their home-based healthcare services [5, 7, 28]. Their Trust Model of service delivery replaced a system of an internal service ordering and delivery mechanism. The model centered service provision decision making on the perceived service needs of service users, and healthcare professionals were given greater authority and responsibility for service delivery. Routines for control and reporting were simplified to ensure integrated services, and interprofessional, autonomous teams were implemented. The model aimed to increase a sense of security, satisfaction and self-determination for service users, increased motivation and job satisfaction for employees, and increased service flexibility, efficiency, and quality. Both examples, as well as the organizational model studied in this paper, have the aim of better performance through giving employees greater autonomy in common, resonating with the HIWP concept found in HRM literature.

### High-involvement work practices and systems

HRM studies have long aimed for identifying HRM practices that may be combined to form high-performance work systems (HPWS). Such systems are a valued goal for policy makers and practitioners alike. The concept has, however, proved difficult to define and operationalize across different organizations, industries, and countries. In a recent review, Boxall et al. [8] suggest that high-involvement work systems (HIWSs) is one model for organizing work that may lead to higher performance and better outcomes.

HIWSs are, firstly, characterized by high-involvement work processes (HIWPs), meaning that employees have high levels of influence over their work processes. This may be in the form of autonomy and control over individual tasks, and/or in the design of how work is organized at the level of teams or workplaces. It implies less control by technology, bureaucratic rules and managerial supervision, and more decision latitude for employees when it comes to choices regarding issues such as working methods, scheduling, the tempo of work, the order in which work tasks are performed, and/or the criteria by which performance is evaluated [8]. The perspective of the HIWP concept stands in opposition to models of work organization such as scientific management and Taylorism. It draws on well-known theories within HRM studies such as the job characteristics model [29], the job demand-control model [30], and the job demands-resources model [31], arguing that increased employee autonomy fosters motivation, engagement, learning and job satisfaction. Indeed, a majority of studies on HIWPs find that these practices have a positive, overall impact on employee outcomes such as job satisfaction, affective commitment, job performance, organizational citizenship behavior, reduced turnover intention, decreased absenteeism and improved work-life balance [8].

**Fig. 2:**
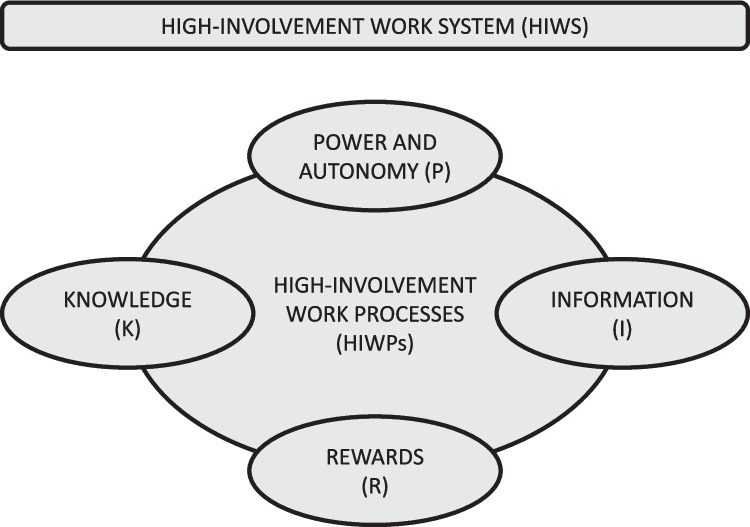
High-involvement work system and elements of high-involvement work processes

However, HIWPs also come with a risk of work intensification rather than beneficial employee empowerment, and intensification may result in employee fatigue, stress and work-life imbalance [32]. Therefore, they need to be combined with an investment in the organization’s human capital in the form of better two-way communication between management and employees, better information, greater training, higher pay and stronger employee voice mechanisms. This combination of HIWPs and investments in human capital is what constitutes a high involvement work *system* (HIWS). It has been suggested that this principles can be summarized in the acronym PIRK [33], denoting the four dimensions of power, information, rewards and knowledge. Figure 2 shows the connections between elements of HIWPs and HIWS.

The first dimension, *workplace power and autonomy*, is in line with a HIWS when employees feel in control over their own work execution and that they get to take part in decisions that impact their work. Further, the dimension of *information* requires that employees perceive communication with management to be effective, the dimension of *rewards* satisfies the HIWS model when employees feel rewarded for their efforts, and the dimension of *knowledge* requires that employees feel they are offered sufficient training and development opportunities [8, 34]. Previous research has found that all four dimensions of the PIRK model must be perceived as present by employees for the expected benefits of HIWPs to materialize [35]. This requires an investment by organizations in mechanisms for consultation and/or negotiation with employees during change efforts and maintaining channels for employee participation in decision-making, employee training, appropriate incentives, and participative management styles [8].

### Tensions and challenges in trust-based management and HIWSs

Previous research has found that autonomy is associated with both job satisfaction and service quality in healthcare organizations [36, 37]. However, new work roles may be challenging to navigate at all organizational levels during the implementation of trust-based models. Bentzen [6] points out that there is a complex systems of interconnected actors who all need to grant each other trust, both in trusting others, and in accepting the invitation of trust. Managers need to open up a space for employee autonomy and professional decision latitude, meaning that they need to step away from detailed control and move towards managing by defining the room of maneuver for employees. At the same time, all actors need to accept greater risk and vulnerability. Managers must accept a certain risk of failures and be willing to allow for problem solving locally. Employees may have a strong interest in being granted greater trust, but this also comes with a risk for them and they need to be willing to accept this new form of trust. Employees who do not perceive themselves to have sufficient competence to handle increased responsibility may be skeptical of trust models. The reports from trust model implementations in Oslo also point out that they involve changes to work roles, and that increased autonomy and responsibility demands an acceptance of risk both at the level of the individual employee and for the system surrounding their work [7, 28].

The literature on HIWSs points to similar challenges. While well-functioning HIWSs may produce gains for both employees and organizations, HIWPs may also increase job demands and have a negative impact on employee well-being [38]. Boxall et al. [8] identify four points of tension within HIWSs. First, the model highlights the importance of achieving internal fit in the combination of HRM practices used in an organization. If greater involvement of employees is implemented to enhance performance, but in combination with practices that simultaneously lead to reduced job security in order to reduce costs, the system will most likely result in unproductive outcomes. Second, increased involvement may lead to work intensification for employees. In order to help employees navigate this tension, supporting front-line managers and investing in their training may enable them to help employees as well as giving the managers a sense of control and self-efficacy. Third, involvement may come in different forms and degrees, meaning that the specific form of involvement implemented in any organization may be perceived as too much involvement, or the wrong kind of involvement by employees. Fourth, and finally, different levels of management may not be aligned and in agreement on the specific regime of involvement implemented, the role of front-line managers may change, and they may not have the support they need in order to handle their new role.

Fundamentally, “high-involvement working is an ongoing experience of high levels of influence over the decisions that affect the work process, identified through individuals’ perceptions of their jobs and their working environment” [39]. In the following, we therefore turn to an analysis of how employees and managers at different levels of the organization in the municipal home-based healthcare services perceived the new organizational model in terms of the dimensions of HIWSs, aiming to shed light on the research question of whether trust reform initiatives may be a strategy for fostering such systems, while also steering clear of the tensions they embody.

## Methods

### Design, data collection and analysis

The research project this article draws its empirical material from was commissioned by the municipality piloting the new organizational model and executed in collaboration with the municipality based on an evaluative trailing research (ETR) design. ETR is a dynamic and flexible way of conducting research-based evaluations, where research activities should provide valuable input to the practice field during change processes. Olsen and Lindøe [40] describe ETR as an approach that “takes a position in between the traditional research ideal of observing and being objective, and the role as a change agent predominant in action research” (p. 372). In our project, the ETR design did not include the research team as active change agents during the design or implementation of the pilot project. Our role was to follow the municipality’s change effort from the sidelines. While preliminary impressions were discussed with pilot project management as it unfolded, our main contribution was in the form of a report delivered at the end of the pilot period, answering research questions that were collaboratively developed with the municipality at the outset of the pilot project (see Appendix 1). This report was then actively used in the process of deciding whether to extend the new model to other home-based care units in the municipality, and to further develop both the model and the change implementation process following the pilot.

The research was conducted in the earliest stages of pilot implementation. The main activities were interviews with key personnel and knowledge exchange between researchers and the involved actors in the pilot project. 20 semi-structured interviews involving a total of 34 informants were conducted. The informants were employees, managers, and team coordinators and supervisors in both healthcare teams (6 interviews) and care service teams (10 interviews), as well as municipal health service managers (4 interviews) at different organizational levels. The selection of informant categories was made by the research group in dialogue with the pilot project’s steering group, which included all the most central stakeholders of the pilot. On an individual level, the informants were recruited by their manager. While this recruitment strategy may propose a risk in terms of bias, the diversity of reflections these informants shared implies that the respondent group represented a balanced set of experiences and opinions. Group interviews were conducted with employees (2-4 informants in each interview), while the other informants were interviewed individually. All interviews were based on a semi-structured thematic guide comprising operationalizations of ETR research questions. The interviews covered topics including participants’ experiences of working under the new organizational model and perceptions of changes brought about by the reform. There were also questions concerning competence and knowledge, trust, motivation, and interprofessional collaboration. Questions were of an open-ended nature, enabling informants to go into detail about what they deemed significant.

In addition to conducting interviews, the research team also followed the work of various groups having a role in running the pilot (i.e., steering group, reference group, and project groups), mainly through examination of meeting minutes, but also occasionally as observers at meetings. Due to the coronavirus pandemic, most meetings were conducted digitally. The data derived from these sources served mainly as supplementary and background information in this paper.

In this paper, we focus on the experiences of managers, registered nurses, healthcare workers and assistants in the new healthcare and care service teams. Although closer muti-professional collaboration between nurses and physical/occupational therapists (who are organized under a different municipal department) was a goal and an integral part of the new organizing model, this aspect is not highlighted in the present paper. The therapists formed a small group of individuals relative to healthcare workers, assistants and registered nurses, and the majority of work in the home-based healthcare services is performed by these three latter groups.

The analysis of interview data was based on template analysis principles [41]. All interviews were recorded. The researchers used interview notes as basis for initial discussions of main impressions and findings, and based on these discussions, the most informative interviews were transcribed. The verbatim transcriptions were thereafter analyzed in detail by use of Nvivo. The text was coded and categorized based on *i)* a predeveloped list based on two of the research questions developed in collaboration with the municipality and the themes in the interview guide related to these, and *ii)* codes and categories that appeared as relevant and important as the analytical process progressed (see Appendix 2), and further *iii)* analyzed by comparing and contrasting findings from steps i) and ii) to the main components of the HIWS model relevant to this study (involvement, power and autonomy, information and knowledge), as well as the known tensions and challenges of this model, as detailed in the theory section above.

## Results

The trailing research on the piloting of the new organizational model for home-based healthcare service delivery [22] found that the model holds potential for realizing the benefits it aims for. However, there were also challenges that need resolving for the model to fulfil this potential. Central elements include clarification of roles and responsibilities for employees and managers, competence specification and development, and development of structures for inter-professional cross-team collaboration and information provision. In this section, we first describe how employees experienced the new organizational model and changes to their work roles in terms of autonomy and involvement, before going on to present an analysis of the wider system of support surrounding the new work roles. In the latter, we focus on the PIRK dimensions of information and knowledge. Rewards were not included as a factor in the organization model pilot, and is therefore omitted from the analysis.

### Employee experiences of the new organizational model as a HIWP: power and autonomy.

#### Healthcare workers and assistants in care service teams

This group experienced the most extensive changes to their professional work tasks, work methods, autonomy and responsibilities in the pilot project, and was particularly exposed to a transition to more involvement. Healthcare workers and assistants did not previously hold independent responsibility for autonomously evaluating and observing changes to the health condition of the service users they visited or for recording changes in their needs. Following the new organizational model, these work tasks were now delegated to the members of care service teams. In addition, service users who did not need care services requiring formal competence at the level of registered nurses would previously still be seen by registered nurses on occasion. However, these service users were now exclusively visited by care service team employees. Thus, healthcare workers and assistants were put autonomously in charge of performing certain tests and presenting test results to other medical personnel for further evaluation when needed. This required always keeping test equipment available in their cars and being able to use the equipment as prescribed.

In addition to changes in tasks and professional responsibility in the work related to direct service user contact, care service team employees were also given more independent administrative responsibilities. This entailed documentation of the service users on different digital platforms. The tasks of collecting and reporting information about services users had for the most part previously been delegated to registered nurses. Another aspect concerning the administrative responsibility was that several healthcare workers were assigned the role as shift coordinators – a role which had also previously been part of the domain of registered nurses. A designated team coordinator held an overall responsibility of team activity but depended on the observations and reports of all team employees as input.

#### Registered nurses in healthcare teams

For registered nurses, their professional work and decision latitude remained mainly as before. However, professional work in direct contact with service users became more concentrated around tasks requiring their formal level of qualification. This included, for instance, tasks related to wound care and palliative care.

Following the delegation of various tasks to the care service teams, it was reported by several registered nurses that they experienced having more time to fulfill professional and administrative office duties, whereas this work had previously been constrained by a lack of time. Many of the interviewees framed this as ‘the invisible work’, and one registered nurse explained: “*I have to say that after we initiated the project we have had more time for this invisible work. We used to be distracted by having to go out and assist with showering or heating up dinners. There used to be more of that. So that is a positive development”.*

Furthermore, the registered nurses reported that they felt their professional competence was more efficiently utilized in the new organizational model, and a sense of mastery and motivation as they had more time to fulfill all aspects of their work. In describing changes in the experience of professional competence, one registered nurse pointed out that *“the day-to-day work has changed for the whole nursing staff in that way. It’s much more specific to our profession. It’s very positive. I think we all agree”.* Several nurses also highlighted that they perceived being trusted by their nearest supervisors to make autonomous, professional decisions in their work with service users, and decisions on the ordering and tempo of office work tasks. The words of a registered nurse vividly illustrate this: *“In terms of trust, it’s a very good thing that we get to decide for ourselves how much time we spend and so on. How much time we spend (on tasks), and being able to plan for how much time to allocate. That we ourselves evaluate needs, what they need help with, and how long it takes.”*

### Employee experiences of the new organizational model as a HIWS: Information and knowledge

#### Information and knowledge needs in new roles

Healthcare workers and assistants in care service teams were given new work roles that implied new responsibilities and new tasks, both of which were experienced as somewhat undefined. For instance, this interviewee from a care service team points out that task allocation and patient responsibilities were not clear: *“Right now, things are unclear. What are our tasks and what are their tasks? (...) Who is responsible for this patient? Is it us or them? I find it a little confusing*.”

In the care service team, variations were observed regarding the perception of the new work roles and responsibilities. Some informants were motivated by the potential for more professional autonomy and involvement. Others were frustrated by what they perceived as a lack of information about what was expected of them and felt insecure about whether their existing knowledge and competence enabled them to sufficiently fulfill their new roles. Employee perceptions varied according to the individual employees’ interest for and previous experience with an autonomous professional focus and administrative work, and with the degree to which they experienced getting professional support and training in new tasks. Employees in the care service teams were given training in new work tasks, sometimes from members of the healthcare teams, but the training was not universally offered ahead of reorganization. Further, access to and training in the use of necessary digital platforms was not offered prior to the reorganization, and routines for the shift coordinator role were not developed and finalized until the new organizational model had been in place for some time.

The degree to which the potential for more fulfilling work for employees and service quality for end users was realized through high involvement depended on the specification and clarity of role definitions, training, and formal support from employees with experience in relevant work tasks. Weakly defined roles and low formal support led to individual variation in both perceptions of job satisfaction and in performance, depending on the previous experience and work relations of individual employees. Front-line managers of both types of teams reported that trusting the care service team employees to make autonomous decisions in their work was an important ideal. However, they were unsure about how much professional independence they should grant employees in a situation characterized by many new and unfamiliar tasks.

Registered nurses reported that they felt they had a responsibility for and were worried about the professional quality of the work, and the situation for employees, in the care service teams. As pointed out above, registered nurses held more clearly defined and formal professional responsibility for service operations in the previous organizational model, whereas the piloted model gave more autonomous responsibility to healthcare workers and assistants. Although registered nurses informally took on responsibility for helping and supervising care service team members when needed, they reported a wish for more information about how these relations were meant to function in practice, and for clearer definitions of the content and limits of their responsibilities. One nurse described this by noting: *“What we have been told is that they (the care service teams) are responsible for testing and evaluating in order for them to develop skills in evaluating whether a patient needs to see a doctor or go to the hospital. But we are to support them and provide some safety. (…) I feel an increase in responsibility, really. Both in a positive and a negative way. Or at least in terms of missing clearer boundaries and a clarification of roles up front.”*

Some of the new care service teams were led by front-line managers with little experience from similar roles, and without registered nurse qualifications. Experienced front-line managers who were qualified in nursing reported feeling responsible for supervising or supporting these managers, their teams, and their employees. As is illustrated by this interviewee, a nurse managing a care service team: *“Right now, as we start out, I feel a heavier responsibility. This also concerns the fact that I am in charge of training three new managers, and I feel a little… I really feel responsible for the other team, for it to function well. The care service team”*. This responsibility was partly formalized as they were asked to take on the task of training new managers, but there was no training ahead of reorganization, and the content and limits of this responsibility was unclear. For instance, there was no information given to experienced front-line managers about their liability in case mistakes were made in the teams led by inexperienced managers.

In summary, we found that all these groups were given new roles, and that without sufficient information and investment in training, i.e. knowledge, they experienced work intensification in the early phases of pilot implementation.

#### Information and knowledge needs for cross-team collaboration

The new organizational model dismantled previous team structures and routines for cross-team collaboration. At the time of study employees and managers in both types of teams were experiencing uncertainties regarding how to achieve necessary practical cross-team collaboration, and knowledge and information exchange.

One aspect of this uncertainty was observed in the process of allocating service users to individual teams. In the earliest stages of the pilot, routines for the allocation of service users were both weak and unclear. In addition, if the conditions or needs of service user changed and they were transferred to a different team, there were insufficient routines for notifying the employees who had cared for them, causing them to worry about whether individual service users were still getting the services they needed. In the allocation process, there was also confusion between teams as to what responsibilities they held vis-a-vis each other. Care service team members were unsure about where to direct questions regarding professional decision-making or practical clarifications, and healthcare team members were unsure about how far-reaching the new responsibilities of healthcare workers and assistants were. Altogether, this caused frustration among employees in the different teams and placed strain on the cross-team collaboration processes.

The role of front-line managers was central in negotiating these issues. However, managers also experienced uncertainty because structural arrangements concerning their new roles, such as clear guidance about the content and limits their responsibilities and routines for cross-team manager and employee interactions, were not settled. It was challenging to deal with the information needs of employees regarding these matters, as they did not have sufficient information themselves. Horizontal and vertical information flow and interaction between managers are significant. A lack of horizontal role clarity among middle managers was experienced as negative for their job mastery, and furthermore weakened their abilities to assure that accurate and consistent (agreed-upon) information was communicated to their employees.

The reorganization created a heightened need for systems and routines ensuring sufficient information about new role expectations, definitions of responsibilities, and task allocation in the collaborative relations between teams, as well as clear structures for information and knowledge exchange across teams in the new organizational model. The general lack of such structures in the early stages of pilot implementation created “noise” within teams and in cross-team relations, and uncertainties for employees as well as managers.

**Fig. 3:**
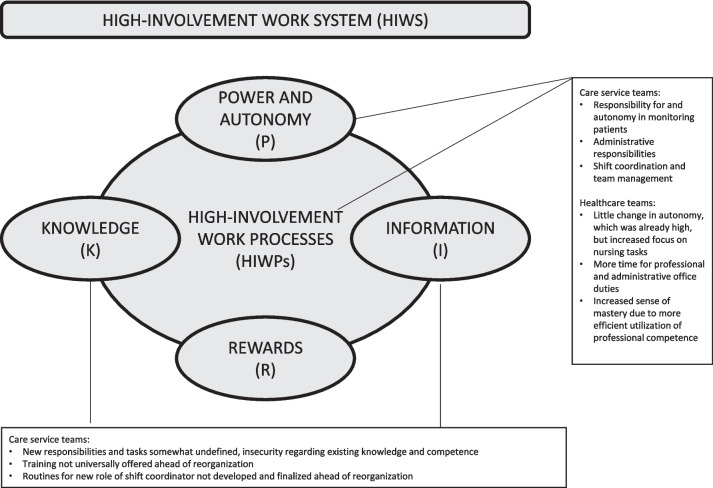
Summary of findings related to elements of high-involvement work processes

The findings related to the HIWP elements of power and autonomy, information, and knowledge are summarized in Fig. 3.

## Discussion and conclusion

Our findings reflect some of the tensions in HIWSs as described by Boxall et al. [8]. In this section, we first discuss one of these points of tension, before briefly outlining other challenges to achieving positive employee and performance outcomes when implementing trust reform initiatives.

The first point of tension is the balancing act between increased work involvement and work intensification [8]. Without sufficient investment in resources such as information and knowledge, the benefits of more power over and autonomy in one’s own work may not materialize. In the case we examined, this balance was not productively achieved in the early stages of pilot implementation. There was a clearly reported need for resources and mechanisms for increased role understanding and role security for employees in both types of teams, and for front-line managers. According to Boxall [8], employees in any organization in which the degree of involvement is altered by reorganization may perceive that the new way of working offers too much, or the wrong kind, of involvement. Research on barriers to implementing innovation in the public sector points to the fact that these barriers may stem from characteristics of the innovation itself, as it may not be compatible with the environment it is introduced to. Barriers may also stem from organizational obstacles, such as the pre-existing structure and culture [26, 42]. In this study, the strongest expressions of insecurity after the reorganization came from employees in the care service teams, which were a particularly diverse group in terms of prior education and experience, age, and preferences regarding the balancing of autonomy against support and security. Introducing new and unfamiliar tasks, more responsibility and stronger independence was a significant break with the professional role that they were accustomed to, and the new role went beyond what some were comfortable with.

Investing in clear information and development of new knowledge through competence building and training for employees ahead of pilot implementation could have ameliorated some of the experiences of intensification. In this respect, the pilot project was challenged by an organizational barrier referred to in the innovation literature as the ineffective administration of process activities [26, 42]. Communication work is clearly challenging in this context due to the nature of home-based healthcare services. Employees spend most of their working hours on the road and in patients’ homes, in addition to working a complex system of shifts, including outside of regular working hours. An active communication and competence building effort was built up during the pilot project period. The project manager was in frequent contact with front line managers and employees, distributed newsletters about the project, and organized courses and other forms of teaching on subjects relevant to the new roles. However, the timeline of and resources allocated to the project did not allow for this to take place prior to implementation.

Role insecurity and insufficient competence for managers may have also fed into role insecurity for employees, as they had less support from their managers than before. Investing in clear and confident leadership in a situation of great change in work tasks and responsibilities for employees, therefore, stands out as something that could have contributed to more security for both managers and employees. This is in line with Bentzen [6] who highlights the fact that trust-based management poses new and unfamiliar requirements for front-line managers, and with Boxall [8] who points to the importance of training and supporting managers in HIWSs so that they can support their staff as well as handle their own role and feel in control of their new situation.

Further, exchange of information and knowledge across teams and professional groups represent important mechanisms for developing job mastery for employees in new work roles involving increased responsibility, autonomy, and task diversity. Both formal (e.g., courses) and informal mechanisms are crucial. Regarding the latter, opportunities for immediate support and feedback depending on the task at hand may be of particular importance in the early phases of such a change process. Boxall et al. [8] also point out that job enrichment and autonomy is just one element in a wider and complex work system. Individual autonomy must be balanced against the performance of interdependent teams, and autonomous teams must be balanced against necessary inter-team coordination and collaboration, which is a significant effort given that coordination between teams may become a major challenge in work systems characterized by high levels of team autonomy [43]. We found in this regard that there is a need for paying attention to the collaborative relations between teams so that the exchange of information is functional, task distribution is optimal, and professional resources are fully utilized to the benefit of both service users and the competence development of employees. This furthermore underlines that building shared understanding across teams is especially important in work environments characterized by complexity and task interdependency [44]. However, recognizing the importance of interdependencies and appreciation of other professionals’ roles and competencies can be challenging [45], which emphasizes the significance of our finding that the work system must include mechanisms contributing to increased understanding of each individual’s work role and their function in the total work system.

We collected data from the pilot project in the early stages of implementation. The organizational model was still under development, and some of the resources needed to deal with the challenges we uncovered were partially being provided as the pilot project progressed. The fact that these resources were not planned for and provided ahead of implementation may be related to one of the barriers to successful trust reform implementation identified by Bentzen [6]. She points out that public service organizations often encounter resource barriers [26, 42] in such implementation processes, meaning that there are limited resources for thorough preparation and involvement of employees in these preparations. Most of our informants reported that they had not been involved in the planning for and development of the new organizational model or its implementation, and they had no clear understanding of its most important goals. In light of previous research, which has highlighted the importance of establishing common understanding [46] early investment in the formation of a consensus of the goals of the model could have contributed to greater ownership of them, to greater engagement in the early stages of implementation, and to resilience in dealing with challenges as they were being worked out and solved. We also know that change processes with real and meaningful employee involvement gives access to the expertise of employees in designing practical solutions that will function in everyday work [47]. This all requires an acknowledgement at all managerial levels of the fact that change processes require allocation of sufficient resources, and a willingness to invest these in order to avoid challenges such as work intensification and insufficient cross-team collaboration after implementation.

As the pilot project came to an end, the municipality concluded that the organizing model was a suitable instrument for achieving the aims they has set out to reach. Notably, the registered nurses in healthcare teams were deemed to have improved their capacity to perform tasks requiring nursing competence, and they were also increasingly able to contribute to the competence development in care service teams. They therefore decided to move on to a full, although step-wise, implementation of the new organizing model to all municipal home-based health care units.

The studied trust reform initiative introduced work organization similar to HIWPs with increased power and autonomy for employees in both types of teams, but other dimensions of HIWSs were underdeveloped at the time of implementation. We believe trust reform initiatives may be a strategy for fostering such systems, while also steering clear of the tensions they embody, if sufficient attention is paid to and resources are allocated to ensuring structures for information exchange and knowledge development in the early phases of implementation, or preferably prior to implementation. Regarding the combination of literature that we have used in this paper, comparing trust based organizational models to the HIWSs model found in the HRM literature, we believe that the literature on HIWSs not only specifies important elements and dimensions of trust based models, but also describes some of the important tensions within them. Therefore, familiarity with the HIWS model and literature could potentially be a useful managerial tool in preparing for and implementing trust reform in healthcare services.

### Supplementary Information

**Additional file 1:  Appendix 1.** Research questions developed in collaboration with the municipality at the outset of the pilot project and ETR process.

**Additional file 2: Appendix 2.** Data analysis.

## Data Availability

The data used and/or analysed during the current study are not publicly available. Data in the form of transcripts (in Norwegian) are available from the corresponding author upon reasonable request.

## Abbreviations

ETR: Evaluative Trailing Research
HIWP: High-Involvement Work Process
HIWS: High-Involvement Work System
HPWS: High-Performance Work System
HRM: Human Resource Management
NPG: New Public Governance
NPM: New Public Management
PIRK: Power, Information, Rewards and Knowledge

## Footnotes

1 This term refers to the Norwegian occupational title of “helsefagarbeider”.
