# Supplementary material for: Reforming for trust and professionalism in municipal healthcare services: implications for human resource management
Source: BMC Health Serv Res. 2023 Sep 14;23:991. doi: 10.1186/s12913-023-10006-8 (PMC10503110; doi:10.1186/s12913-023-10006-8)
Supplement: Supplementary file 1 — Additional file 1: Appendix 1. Research questions developed in collaboration with the municipality at the outset of the pilot project and ETR process. [file 12913_2023_10006_MOESM1_ESM.docx]

# APPENDIX 1

## Research questions developed in collaboration with the municipality at the outset of the pilot project and ETR process

### Main research question

What are the central organizational barriers and opportunities in the implementation of healthcare and care service teams in the municipality?

### Sub-questions

1. Does the new model lead to changes in how employees experience their work (professionality, responsibility, mastery, trust and motivation)?

2. Does the new model lead to changes in collaboration and coordination, both within and between professional groups?

3. Does the new model lead to changes in how the employees execute their work (more/less methodical and goal oriented)?

4. Do the research results point to necessary changes to the piloted model?

5. Which conditions must be considered in the further implementation of the trust and professionalism reform in the municipality?

6. Which themes and research questions should be prioritized in a larger research projects in the future regarding organization, financial considerations, and the involvement of patients and next of kin?

7. What expertise and which actors should be involved (nationally and internationally)? How could and should such a research project be organized?
