# Supplementary material for: Reforming for trust and professionalism in municipal healthcare services: implications for human resource management
Source: BMC Health Serv Res. 2023 Sep 14;23:991. doi: 10.1186/s12913-023-10006-8 (PMC10503110; doi:10.1186/s12913-023-10006-8)
Supplement: Supplementary file 2 — Additional file 2: Appendix 2. Data analysis. [file 12913_2023_10006_MOESM2_ESM.docx]

# APPENDIX 2

## Data analysis

### Relevant research questions from the ETR-project, initial codes (step *i*) and emerging themes (step *ii*)

| Relevant research questions from the evaluative trailing research project | Initial codes (first step of analysis) | Emerging themes (second step of analysis) |
| --- | --- | --- |
| Does the new model lead to changes in how employees experience their work (professionality, responsibility, mastery, trust and motivation)? | Professionality | - Increased professional demands in patient care - Increased professional demands in administrative tasks - Professional demands unchanged - Work tasks tailored more specifically to competence of each professional group - More time for administrative tasks |
|  | Responsibility | - Increased responsibility for patient care - Increased responsibility for administrative tasks - Responsibility in patient care unchanged - Responsibility in administrative tasks unchanged - Responsibility more focused - Increased (informal) responsibility for the work delegated to other teams |
|  | Trust | - Increased experience of being trusted to make professional decisions - Increased experience of being trusted to manage time spent on tasks - Doubts about whether teams should be trusted with increased professional demands and responsibilities |
|  | Mastery | - Mastery as a result of new demands and responsibilities - Mastery as a result of more time to complete professional tasks - Insecurity as a result of new demands and responsibilities - Insecurity as a result of unclear demands and responsibilities |
|  | Motivation | - Increased demands and responsibility as motivational - Increased demands and responsibility as demotivating |
| Does the new model lead to changes in collaboration and coordination, both within and between professional groups? | Internal team collaboration /  coordination | - Challenges regarding the merger of work routines from different pre-existing units into new teams - Diverse group of employees in care service teams |
|  | Cross-team collaboration / coordination | - Erosion of pre-existing collaboration between a) nurses and b) healthcare workers and assistants - Different types of teams have separate arenas for coordination work - Lack of systems for handling cross-team interfaces - Unclear responsibilities for supporting other teams |
